# Pulmonary Immune-Compartment-Specific Interferon Gamma Responses in HIV-Infected Individuals with Active Tuberculosis (TB) in an Area of High TB Prevalence

**DOI:** 10.1155/2012/308473

**Published:** 2012-06-21

**Authors:** S. Buldeo, D. M. Murdoch, M. S. Suchard

**Affiliations:** ^1^Department of Molecular Medicine and Haematology, Faculty of Health Sciences and National Health Laboratory Service, University of The Witwatersrand, Johannesburg, South Africa; ^2^Division of Pulmonology and Critical Care Medicine, Department of Medicine, Duke University, Durham, NC 27708, USA

## Abstract

There is a paucity of data on the pulmonary immune-compartment interferon gamma (IFN*γ*) response to *M. tuberculosis*, particularly in settings of high tuberculosis (TB) prevalence and in HIV-coinfected individuals. This data is necessary to understand the diagnostic potential of commercially available interferon gamma release assays (IGRAs) in both the pulmonary immune-compartment and peripheral blood. We used intracellular cytokine staining by flow cytometry to assess the IFN*γ* response to purified protein derivative (PPD) and early secretory antigen 6 (ESAT6) in induced sputa (ISp) and blood samples from HIV-infected, smear-negative, TB suspects. We found that individuals with active TB disease produced significantly less IFN*γ* in response to PPD in their induced sputa samples than individuals with non-active TB (control group). This difference was not reflected in the peripheral blood, even within the CD27− CD4+ memory T lymphocyte population. These findings suggest that progression to active TB disease may be associated with the loss of IFN*γ* secretion at the site of primary infection. Our findings highlight the importance of studying pulmonary immune-compartment *M. tuberculosis* specific responses to elucidate IFN*γ* secretion across the spectrum of TB disease.

## 1. Introduction

TB remains one of the great killers despite adequate chemotherapeutic agents and a vaccine. The current tuberculosis epidemic is fuelled by numerous factors, with human immunodeficiency virus (HIV) associated immunosuppression [1] and the lack of accurate diagnostic assays [2] being two major drivers of the epidemic. HIV and TB-coinfected individuals present a diagnostic challenge as they often have atypical clinical and radiological features, as well as paucibacillary disease with negative microbiological tests [3]. Diagnostic tools that do not rely on the direct detection of mycobacteria are therefore required. Tools based on the detection of *M.  tuberculosis* antigen-specific immune responses are good candidates [4]. 

The T-SPOT.TB and QuantiFERON-TB (QFN-TB) assays are commercially available enzyme-linked immunosorbent spot (ELISpot) and enzyme linked immunosorbent assays (ELISAs) that detect IFN*γ* released by *M.  tuberculosis* antigen-specific cells. These assays are the *in vitro* alternative to the widely used tuberculin skin test (TST) and offer higher *M.  tuberculosis* specificity [5]. These IFN*γ* release assays (IGRAs) have shown limited use in high prevalent settings, as they are unable to differentiate between active and latent TB. Better characterisation of *M.  tuberculosis* antigen-specific IFN*γ* responses may improve understanding of the complex immune response in TB and interpretation of IGRAs.

In contrast to these commercial assays, IFN*γ* detection by flow cytometry has the advantage of identifying the specific cell population responsible for the cytokine production and better sensitivity [6]. Utilising flow cytometry, Streitz et al. suggested that active TB could be differentiated from latent TB if IFN*γ* was detected from CD4+ memory T lymphocytes that expressed a CD27− phenotype [7]. 

Other studies [4, 8] have also shown that the use of pulmonary-based samples, rather than blood, are capable of differentiating active from latent TB infection. Pulmonary compartment-specific samples are advantageous over blood samples as the proportion of *M.  tuberculosis* antigen*-*specific lymphocytes is higher, the sample is less affected by BCG vaccination, the sample can be split for microbiological assessment, and it is potentially less affected by HIV coinfection [4, 8]. Furthermore, sputum induction is a simple noninvasive procedure that can easily be performed in an outpatient department [4] and shows immune responses that are comparable to bronchoalveolar lavage (BAL) [9, 10].

In this study, we investigated IFN*γ* responses to *M.  tuberculosis* antigens in the pulmonary immune-compartment and peripheral blood of HIV-infected individuals suspected of having active TB, with initial sputum smears negative for acid fast bacilli (AFB). Using flow cytometry, the primary aim was to compare the *M.  tuberculosis* antigen*-*specific IFN*γ* responses between blood and ISp samples. The secondary aim was to compare *M.  tuberculosis* antigen*-*specific IFN*γ* responses between the two defined patient groups, that is, individuals with active TB (subsequent smear or culture positive from induced sputa or further samples) and non-active TB (remain culture and smear negative) in an area of high TB prevalence, to determine whether this approach could be pursued to diagnose active TB in HIV-infected patients. After intracellular cytokine staining and detection by flow cytometry, we observed higher IFN*γ* response in sputum than blood. Counterintuitively, we found significantly lower IFN*γ* secretion in response to PPD in the pulmonary immune-compartment of the active TB group than the non-active TB group. This difference however, was not reflected in the peripheral blood, neither in the total lymphocyte, CD4+ T lymphocytes nor in the CD27− CD4+ memory T-lymphocyte population. The detection of IFN*γ* from ISp samples by flow cytometry is however, impractical for large-scale use and does not have sufficient sensitivity and specificity to be employed as a routine diagnostic assay.

## 2. Materials and Methods

### 2.1. Study Population

HIV-positive adult (>18 years) patients undergoing diagnostic workup for suspected TB “TB suspects”, after producing two sputum smear samples negative for AFB, were enrolled from the Helen Joseph Hospital or Charlotte Maxeke Johannesburg Academic Hospital between June 2010 and March 2011. TB suspects included patients with symptoms suggestive of tuberculosis, such as persistent cough of longer than two-week duration, weight loss, or night sweats with no alternative diagnosis found by routine workup at a tertiary health care centre. Exclusion criteria included anti-tuberculosis chemotherapy for more than one week, a history of asthma or chronic obstructive pulmonary disease, administration of a concurrent immunosuppressant regimen (e.g., corticosteroid therapy), and a recent life-threatening event. Ethical approval was obtained from the University of Witwatersrand, Human Research Ethics Committee.

### 2.2. Sample Collection

Five millilitres (mL) of venous blood was collected into an acid citrate dextrose anticoagulated tube. Sputum was induced after addition of 20 mL of 5% hypertonic saline to a nebuliser mask, which was delivered at a rate of six litres of oxygen per minute. The induction continued for 15–20 minutes. Three to five millilitres of sputum was collected into a sterile container.

### 2.3. Sample Processing

Blood and ISp samples were processed simultaneously and within two hours of collection. Sputolysin (Caldon Biotech, USA) was added to sputum samples to digest the mucus. These samples were rolled at room temperature for 15 minutes and then filtered with a 100 *μ*m filter. Peripheral blood mononuclear cells (PBMC) were isolated by density-gradient centrifugation using standard histopaque methods (Sigma Aldrich, USA). Following preparation and isolation, ISp and PBMC samples were stimulated with 10 *μ*g/mL PPD (Statens Serum Institut, Denmark), 10 *μ*g/mL ESAT6 (Statens Serum Institut, Denmark), or 1 *μ*g/mL Staphylococcal enterotoxin B (SEB)(Sigma Aldrich, USA) which served as the positive control. Antigenic stimulation was omitted from the negative control. Brefeldin A (0.5 *μ*L/mL, Sigma Aldrich) a cytokine secretion inhibitor and 1 *μ*L/mL each of costimulatory molecules CD28 and CD49d (Becton Dickinson, USA) were immediately added to all cultures. The cells were incubated in 5 mL polypropylene (BD Biosciences, USA) tubes with tissue culture medium (RPMI supplemented with 1 *μ*L/mL each of penicillin-streptomycin and glutathione from Sigma Aldrich, USA) for 18 hours at 37°C in a 5% CO_2_ incubator. One hundred microlitres of ethylenediamine tetraacetic acid (EDTA) (BD Biosciences, USA) was added to detach any adherent cells. Cells were stained for surface markers CD4 PerCp-Cy5.5 (BD Biosciences 341654) and CD27 APC (BD Biosciences, 558664) by incubating for 20 minutes in the dark and at room temperature. Following surface staining, cells were permeabilised according to the manufacturer's protocol (Cytofix/Cytoperm BD Biosciences, USA, 554723) for 15 minutes in the dark and stained intracellularly for CD3 FITC (BD Biosciences, 55918) and IFN*γ* PE (BD Biosciences, 559327). Samples were immediately acquired using the LSRII flow cytometer (BD Biosciences).

### 2.4. Sample Analysis

Samples were analysed using FlowJo software (TreeStar, USA). The flow cytometry panel was optimized prior to commencing the study. Fluorescence minus one experiments were used to minimize spectral overlap. In addition to daily instrument quality control, one-peak beads (BD Biosciences) were used to standardise fluorescence values in each channel over time and compensation was set daily using BD compensation beads. Sample acquisition was stopped when the cellular events' rate dropped to less than one hundred events so that the full volume of the sample was analysed.

**Figure 1:**
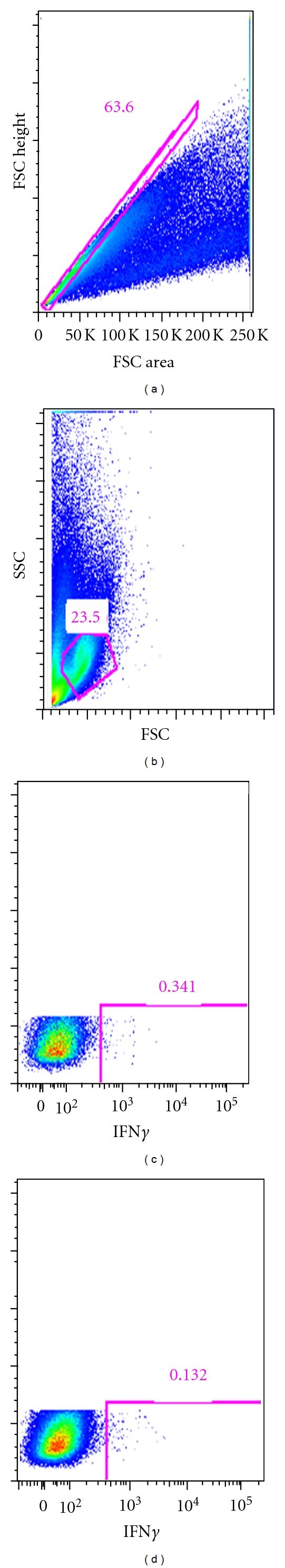
Flow cytometry dot plots showing gamma interferon production from the total sputum lymphocyte population. (a) shows exclusion of doublets by gating cells with proportional forward scatter area (*x*-axis) against forward scatter height (*y*-axis). (b) shows gating of total lymphocytes according to forward scatter (*x*-axis) and side scatter (*y*-axis). (c) shows the gamma interferon produced from total lymphocytes after stimulation with PPD. The position of the gate was determined in the unstimulated control tube (d) and copied into the antigen stimulated tubes.

**Figure 2:**
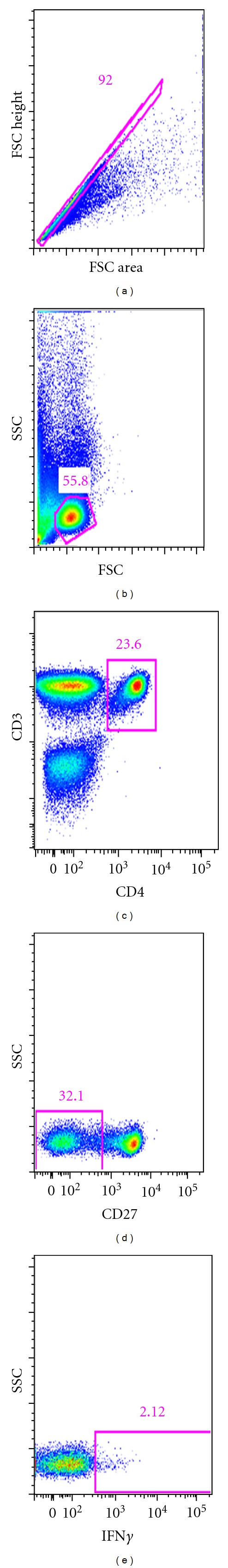
Flow cytometry dot plots showing gamma interferon production from the CD27− CD4+ memory T lymphocyte population. (a) shows exclusion of doublets by gating cells with proportional forward scatter area (*x*-axis) against forward scatter height (*y*-axis). (b) shows gating of total lymphocytes according to forward scatter (*x*-axis) and side scatter (*y*-axis). (c) shows the CD3+ and CD4+ coexpressing lymphocytes. (d) comprises only CD3+ CD4+ lymphocytes and shows the CD27− (CD27 on x-axis and SSC on y-axis) CD3+ CD4+ lymphocytes. (e) shows the percentage of IFN*γ* secreting memory CD27− CD4+ memory T lymphocytes. The position of the gate was determined in the unstimulated control tube and copied into the stimulated tubes.

The frequency of IFN*γ* secreting lymphocytes for each target population was determined utilising a sequential gating strategy (Figures 1 and 2). A forward scatter (FSC) area and FSC height plot were used to exclude doublets or clumps of cells. Lymphocytes were identified from their characteristic cluster on side scatter (SSC) and FSC area. After gating on the lymphocyte population, CD3+ and CD4+ co-expressing lymphocytes were used to identify the CD4+ T lymphocytes. Gating on the CD3+ CD4+ lymphocytes with dim to absent expression of CD27 identified the CD27− CD4+ memory T lymphocytes (Figure 2). Unlike blood, the CD4+ and CD27− CD4+ memory T lymphocyte from ISp samples were too small for reliable interpretation of IFN*γ* secretion. IFN*γ* secretion from sputum samples was measured as a frequency of total lymphocytes (Figure 1). Gates for IFN*γ* positivity were set according to the unstimulated sample.

### 2.5. Definition of Patient Groups

All participants included in this study were HIV-positive individuals who were suspected of having active TB but had negative sputum smear microscopy results and are referred to as “TB suspects.” Suspicion of TB was made by the treating clinician, prior to referral to study investigators. Criteria for “TB suspect” included persistent cough, loss of weight, or night sweats with no alternate diagnosis. A microbiological classification was used to assign final diagnosis. Individuals who, on subsequent investigation, developed positive microscopic and culture results for *M.  tuberculosis* were classified as “active TB.” Individuals who, on subsequent investigations, remained microscopy and culture negative for *M.  tuberculosis* were classified as “non-active TB.” Since a microbiological classification was employed, the non-active TB group potentially included latently infected individuals, in addition to those without TB infection or exposure. There may also have been individuals who were misclassified as non-active TB owing to the lack of sensitivity of microscopy and culture assays.

### 2.6. Statistical Analysis

Data was summarized using descriptive nonparametric statistics. The Mann-Whitney test was used to compare IFN*γ* production between blood and Isp samples, as well as the active and non-active TB groups. Eleven sputum samples were excluded, and Fischer's exact test was used to compare this subgroup to the participants included in the study. Owing to a paucity of cellular content, IFN*γ* production was quantitated only from the total lymphocyte population in sputum samples but neither from the CD4+ T lymphocytes nor the CD27− CD4+ memory T lymphocytes. A value of *P* < 0.05 was considered significant. A receiver operating curve was used to establish sensitivity and specificity. GraphPad Prism version 5 was used for statistical analyses. Data was analyzed as raw values and with the unstimulated background IFN*γ* secretion subtracted from the stimulated values. Only the raw data has been reported. The subtracted data showed similar trends.

## 3. Results

### 3.1. Study Population

Thirty-one patients were enrolled and had peripheral blood available for analysis. One patient was unable to expectorate following sputum induction. Despite optimization of sputum processing techniques, 11 sputum samples were excluded due to assay-related factors, which included poor lymphocyte viability and the presence of nonspecific monoclonal binding. Only 19 ISp samples were suitable for sputum analysis. Of the 19, 9 were classified as active TB and 10 as non-active TB. Of the 31 patients who had blood analyzed, 17 patients were classified as active and 14 as non-active TB. PCR analysis (Genotype MTBDR, Hain Lifesciences, Germany) of all positive results confirmed the isolates as *M.  tuberculosis.*

### 3.2. Sample Demographics

**Figure 3:**
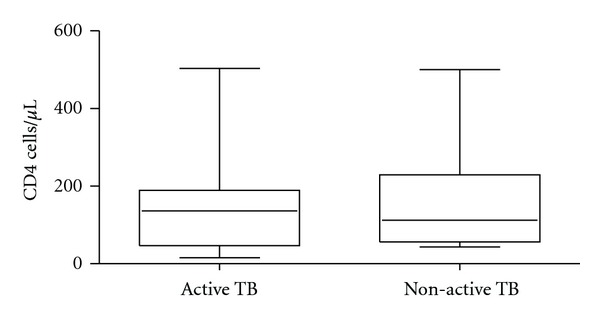
CD4 count in the active and non-active TB groups.

Thirty-five percent of the sample population was on antiretroviral therapy, and the sample included 48% female. The median age was 37 years old (range, 17 to 61 years), and the median CD4 count was 117 cells/*μ*L (range, 15 to 503 cells/*μ*L). There were no significant differences between the active and the non-active groups regarding age and CD4 counts (Figure 3), but the active TB group comprised more males than the non-active TB group (10 in active versus 6 in non-active). The age and CD4 count of the patients that were excluded for poor sputum quality, did not differ from the patients that were included.

### 3.3. Quantitation of *M.  tuberculosis*-Specific IFN*γ* Secreting Lymphocytes in Blood and Sputum

All participants produced IFN*γ* in response to the positive control, SEB as well as the *M.  tuberculosis* antigens.

#### 3.3.1. IFN*γ* Secretion from Sputum Is Higher than Blood

**Figure 4:**
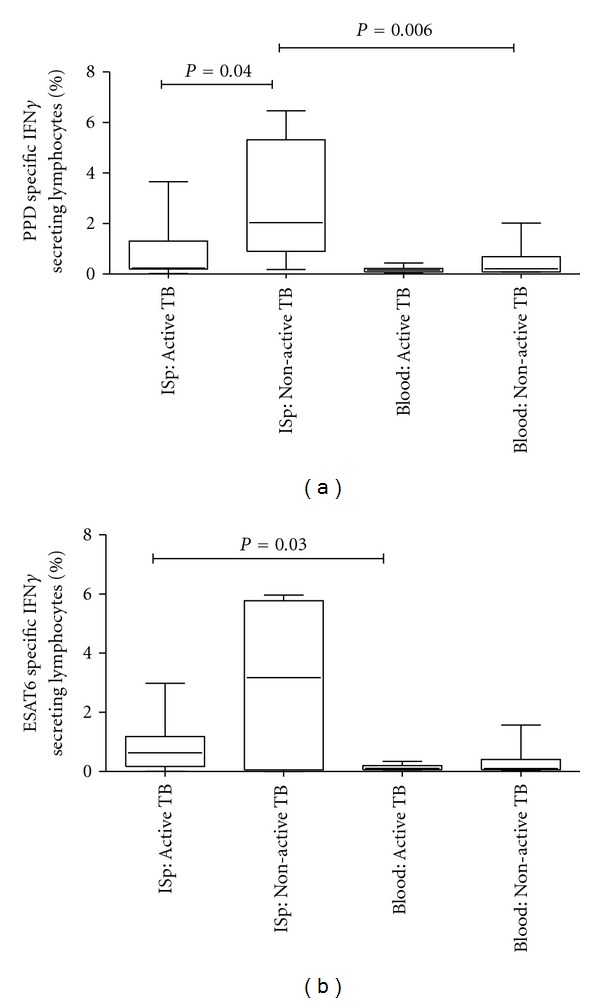
PPD (a) and ESAT6 (b) specific IFN*γ* secreting lymphocytes in blood and sputum of patients with active and non-active TB. Sputum PPD-specific lymphocytes were significantly lower in the active TB group compared to the non-active TB group (*n* = 19). *M.  tuberculosis* antigen-specific lymphocytes were more frequent in sputum compared to blood.

The percentage of IFN*γ* secreting lymphocytes, as a frequency of total lymphocytes, from blood and sputum were compared to determine whether the pulmonary lymphocytes produced significantly more IFN*γ*. Only blood samples with corresponding sputum samples were used in this analysis (*n* = 19) and are represented in Figures 4(a) and 4(b). The median percentages of ESAT6-specific IFN*γ* secreting lymphocytes in blood and sputum were 0.10% and 0.64%, respectively, in the active TB group and 0.10% and 3.18%, respectively, in the non-active TB group. The median PPD-specific IFN*γ* secreting lymphocytes in blood and sputum were 0.15% and 0.23%, respectively, in the active TB group and 0.21% and 2.04% in the non-active TB group. Thus, the percentage of *M.  tuberculosis*-specific IFN*γ* secreting lymphocytes was higher in sputum than blood. The difference between blood and sputum was significant in the active TB group when stimulated with ESAT6 (*P* = 0.03; Figure 4(b)) but in the non-active the difference was significant with PPD stimulation (*P* = 0.006; Figure 4(a)). 

#### 3.3.2. HIV Infected Individuals with Active TB Produce Less PPD-Specific IFN*γ* in Induced Sputa Than the HIV Infected Non-Active TB Group

In the induced sputum samples, PPD stimulation resulted in significantly less IFN*γ* secretion in the active TB than the non-active TB group (median 0.23% versus 2.04%, *P* = 0.04; Figure 4(a)). Stimulation with ESAT6 showed a similar trend of lower ESAT6-specific IFN*γ* secretion in active TB (median, 0.64%) than the non-active TB group (median 3.18%) but did not achieve statistical significance.

#### 3.3.3. *M.  tuberculosis* Antigen-Specific IFN*γ* Responses in Blood Failed to Differentiate the Active TB Group from the Non-Active TB Group

**Figure 5:**
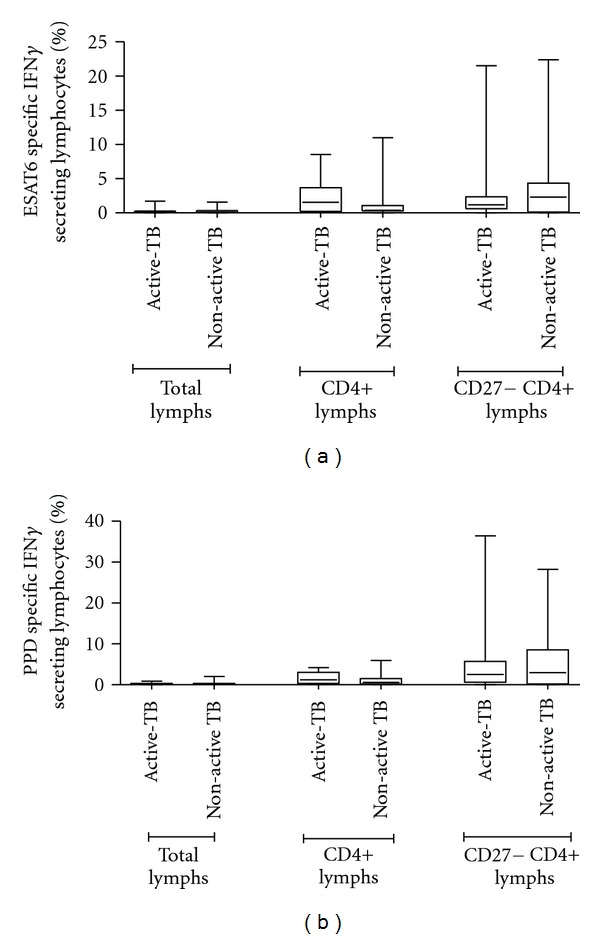
ESAT6 (a) and PPD-specific (b) IFN*γ* secreting total, CD4+ and CD27− CD4+ memory blood T lymphocytes in active and non-active TB groups. No statistical differences were noted between the two TB groups for any of the lymphocyte populations analyzed. Blood, *n* = 31.

In blood, the percentage of neither ESAT6 nor PPD-specific IFN*γ* secreting lymphocytes (total, CD4+ T lymphocytes or CD27− CD4+ T memory lymphocytes) was statistically different when the active and non-active TB groups were compared (Figure 5). The median percentage ESAT6-specific IFN*γ* secreting total lymphocytes was 0.12% versus 0.09% (*P* = 0.65), 1.54% versus 1.07% (*P* = 0.35) for CD4+ T lymphocytes, and 1.17% versus 2.3% (*P* = 0.81) for CD27− CD4+ memory T lymphocytes for the active TB and non-active TB groups, respectively. The median PPD-specific IFN*γ* secretion was 0.15% versus 0.20% (*P* = 0.37) for total lymphocytes, 1.20% versus 0.58% (*P* = 0.33) for CD4+ T lymphocytes, and 2.50% versus 2.96% (*P* = 0.98) for the CD27− CD4+ memory T lymphocytes in the active and non-active TB groups, respectively.

### 3.4. Diagnosis of Active TB Using PPD-Specific IFN*γ* from Induced Sputa 

**Figure 6:**
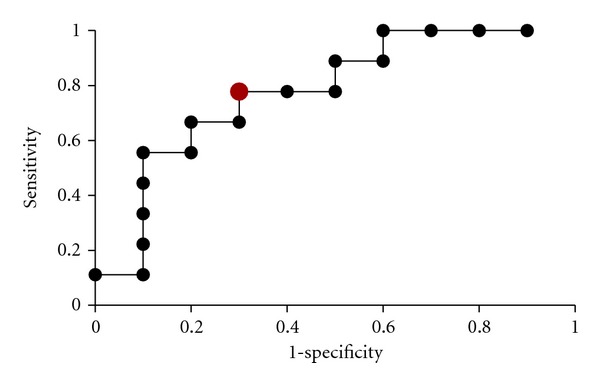
ROC curve analysis of diagnostic accuracy of sputum PPD-induced IFN*γ*. The sensitivity and specificity for discriminating active TB from non-active TB, at a diagnostic threshold of PPD-induced gamma interferon production by 1.2% of total lymphocytes (larger red point), were 78% and 70%, respectively, yielding positive and negative predictive values of 70% and 78%.

A receiver operating curve (ROC) (Figure 6) was generated to calculate the diagnostic accuracy of sputum PPD-specific IFN*γ* for diagnosis of active tuberculosis. A frequency of PPD-specific IFN*γ* secreting lymphocytes at a threshold of 1.20% had a sensitivity of 78% (95% CI, 40–97%) and specificity of 70% (95% CI, 35–93%) to discriminate active TB from non-active TB. The area under the curve was 0.78. The positive and negative predictive values were 70% and 78%, respectively.

## 4. Discussion

Cytokine responses are not well characterised in HIV-infected individuals, in high TB prevalence settings, or at the site of primary TB infection. This immunological study of HIV-infected individuals from a high TB burden area reveals that *M.  tuberculosis* antigen*-*specific IFN*γ* responses differ between the pulmonary and blood immunological compartments.

We found that the frequency of *M.  tuberculosis* antigen-specific IFN*γ* secreting lymphocytes in sputum was higher than that of blood, in correspondence with the findings of others [4, 8]. This finding most likely reflects the potentially stronger immune response at the primary site of infection, the lung.

The frequency of PPD-specific sputum lymphocytes was found to be significantly lower in the active TB group than the non-active) TB group. The reason for this is uncertain but either reflects the diminishing IFN*γ* response as one progresses to active disease or, conversely, a distinct sub-group of patients with active TB but negative microbiological results. It is unlikely that BCG vaccination is responsible for the difference in IFN*γ* secretion noted between the two groups, as the lung functions as a distinct immunological compartment and BCG vaccination has been shown not to interfere with PPD responses from this site [11]. Furthermore, participants from both groups would have received BCG vaccination as it is routinely administered at birth in South Africa. We believe that the associated reduced IFN*γ* secretion, as one progresses to active TB, best accounts for our finding. However, our observation of lower IFN*γ* secretion in the active TB group is in contradiction to a previous report which analysed immune-specific TB responses from induced sputa by flow cytometry [4]. This study was conducted in an area of low TB prevalence and included only three individuals with latent TB infection. Notably, latent tuberculosis infection has been shown to produce higher frequencies of *M.  tuberculosis*antigen-specific IFN*γ* secreting lymphocytes than active disease [12, 13]. Our findings are also in keeping with the documented importance of IFN*γ* in combating TB in human models [14, 15]. Harari et al. [13] analysed the immune responses in individuals with active and latent TB infection and concluded that individuals with latent TB exhibited polyfunctional T lymphocyte immune responses, which included IFN*γ* production, whereas patients with active TB possessed more monofunctional immune responses primarily characterized by exclusive TNF*α* production. Additionally, a study which characterised the CD4+ and CD8+ IFN*γ* responses in patients with active and latent TB infection after stimulation with either PPD or ESAT6 concluded that PPD stimulation in latent TB produced the highest frequency of IFN*γ* cells [16]. These findings are similar to our results, as they show diminished IFN*γ* production as individuals progress to active TB disease.

*M.  tuberculosis* antigen*-*specific CD27− CD4+ memory T lymphocytes have been proposed as a quick *blood*-based tool to diagnose active TB [11, 17]. Streitz et al. [7] showed that PPD-specific blood CD27− CD4+ memory T lymphocytes served as a marker for active TB (sensitivity 100% and specificity of 85%). However, the current study did not detect any difference in antigen-specific CD27− CD4+ memory T lymphocytes between the active and non-active TB groups. This discrepancy likely reflects the differences in study populations. All participants in the current study were HIV infected, which can alter CD27 expression and subsequent proliferation of antigen-specific CD4+ lymphocytes [18]. In addition, HIV and TB coinfection is associated with paucibacillary disease [19], and the loss of CD27 expression on CD4+ cells may be linked to the bacterial burden of the disease in the lung [7]. Furthermore, a recent study showed that the reduced expression of CD27 on *M.  tuberculosis*antigen*-*specific CD4+ cells correlated better with persistent active tuberculosis rather than newly diagnosed active tuberculosis [17]. All participants included in the active TB group were considered individuals with newly diagnosed active TB, rather than persistent active disease. 

Regarding the diagnostic utility of this assay, we found that optimal sensitivity and specificity of PPD induced IFN*γ* production to diagnose microbiological proven tuberculosis, was achieved at a threshold of 1.20% of total sputum lymphocytes and yielded a positive and negative predictive value of 70% and 78%, respectively. However, similar to another study, which used ELISpot to detect *M.  tuberculosis*antigen*-*specific response in ISp samples [20], we found that sputum-based immunological assays are unlikely to be useful in the routine diagnostic setting. The impracticalities of this method include the logistical difficulties involved in the requirement for processing sputum within two hours, and despite meeting this requirement, samples often displayed a paucity of cellular content. A viability assessment using the trypan blue method [20] may have been of value in excluding unsuitable sputum samples prior to processing. Furthermore, the method would have to compete with newly introduced molecular testing method (Gene Xpert, Cepheid) that offers comparable sensitivity and specificity for sputum smear negative samples, with the additional advantages of being more robust, less labour intensive system, and the benefit of drug susceptibility testing [21]. 

Nonetheless, our findings highlight the importance of studying compartment-specific immune responses in elucidating TB pathogenesis. Detailed immunological profiling of such compartmental responses across the spectrum of TB disease will yield valuable insight into the application and interpretation of IGRAs and new diagnostic methods in TB suspects. 

Our study had the following limitations. First, the effect of latent tuberculosis infection in the non-active TB group cannot be quantified, as latency was not diagnosed. Accurate data on the prevalence of latent tuberculosis in South Africa is not available but ranges from 55 to 88% according to data published by local investigators [22–24]. Second, BCG vaccination status was not documented, as patient self-reporting for a vaccine administered at birth is unreliable. However, since BCG vaccination at birth is part of the South African immunisation schedule, it is suggested that most, if not all, participants were vaccinated. Third, the total number of sputum samples was limited due to quality and technical processing issues mentioned above. However, the exclusion of sputum sample was performed independently of the microbiological results. Finally, the sensitivity of smear microscopy and culture, even on ISp, is not 100%; hence, given the limited sample size, misclassification may be crucial. The Xpert MTB/RIF (GeneXpert, Cepheid) was not routinely performed at the time of this study, and inclusion of the Xpert MTB/RIF (GeneXpert, Cepheid) would have improved reliability of the sputum results and patient classification.

Strengths of this study include the determination of *M.  tuberculosis* antigen-specific immune responses in the pulmonary compartment of HIV infected individuals rather than peripheral blood alone, and the inclusion of a diagnostically relevant control group comprising HIV infected TB suspects. 

## 5. Conclusion

In an area with a high TB prevalence, HIV and TB coinfected individuals produce significantly less pulmonary PPD-specific IFN*γ* than HIV-infected patients with non-active TB. This possibly reflects the spectrum of IFN*γ* secretion as an individual progresses from control of latent infection to active TB disease. These findings were not reproduced in blood even after gating on the CD27− CD4+ memory T lymphocyte population and differ from findings in areas of low TB prevalence.
